# Management of aortic disease in children with *FBN1*-related Marfan syndrome: A joint statement from the paediatric subgroup of the European Reference Network of Vascular Diseases (VASCERN, Heritable Thoracic Aortic Disease working group) and the Association for European Paediatric and Congenital Cardiology (AEPC)

**DOI:** 10.1093/eurheartj/ehae526

**Published:** 2024-09-09

**Authors:** Laura Muiño-Mosquera, Elena Cervi, Katya De Groote, Wendy Dewals, Zina Fejzic, Kalliopi Kazamia, Sujeev Mathur, Olivier Milleron, Thomas S Mir, Dorte G Nielsen, Michal Odermarsky, Anna Sabate-Rotes, Annelies van der Hulst, Irene Valenzuela, Guillaume Jondeau

**Affiliations:** Department of Paediatrics, division of Paediatric Cardiology, Ghent University Hospital, C. Heymanslaan 10, Ghent 9000, Belgium; Center for Medical Genetics, Ghent University Hospital, Ghent, Belgium; Inherited Cardiovascular Diseases Centre, Cardiology, Great Ormond Street Hospital, London, United Kingdom; Department of Paediatrics, division of Paediatric Cardiology, Ghent University Hospital, C. Heymanslaan 10, Ghent 9000, Belgium; Department of Paediatrics, division of Paediatric Cardiology, Antwerp University Hospital, Antwerp, Belgium; Department of Paediatrics, division of Paediatric Cardiology, Radboud University Medical Centre, Nijmegen, The Netherlands; Department of Paediatric Cardiology, Stockholm-Uppsala, Karolinska University Hospital, Stockholm, Sweden; Department of Women’s and Children’s Health, Karolinska University Hospital, Stockholm, Sweden; Department of Cardiovascular Imaging, Guy’s and St Thomas Hospital, London, United Kingdom; Centre de réference pour le syndrome de Marfan et apparentés, Department of Cardiology, Bichat Claude Bernard Hospital, Université Paris Cité, INSERM U1148, Paris, France; Childrens Heart Centre, Paediatric Cardiology, University Clinics Hamburg, Hamburg, Germany; Department of Cardiology, Aarhus University Hospital, Aarhus, Denmark; Children Heart Centre, Skane University Hospital, Lund, Sweden; Department of Paediatric Cardiology, Hospital Vall D’Hebron, Barcelona, Spain; Department of Paediatrics, Division of Paediatric Cardiology, Amsterdam University Medical Centre, Amsterdam, The Netherlands; Department of Clinical and Molecular Genetics, Hospital Vall d’Hebron, Barcelona, Spain; Centre de réference pour le syndrome de Marfan et apparentés, Department of Cardiology, Bichat Claude Bernard Hospital, Université Paris Cité, INSERM U1148, Paris, France

**Keywords:** Marfan syndrome, Aortic disease treatment, Aortic imaging, Exercise recommendation, Children

## Abstract

Marfan syndrome (MFS) is a hereditary connective tissue disorder with an estimated prevalence of 1:5000–1:10 000 individuals. It is a pleiotropic disease characterized by specific ocular, cardiovascular, and skeletal features. The most common cardiovascular complication is aortic root dilatation which untreated can lead to life-threatening aortic root dissection, mainly occurring in adult patients. Prompt diagnosis, appropriate follow-up, and timely treatment can prevent aortic events. Currently there are no specific recommendations for treatment of children with MFS, and management is greatly based on adult guidelines. Furthermore, due to the scarcity of studies including children, there is a lack of uniform treatment across different centres. This consensus document aims at bridging these gaps of knowledge. This work is a joint collaboration between the paediatric subgroup of the European Network of Vascular Diseases (VASCERN, Heritable Thoracic Aortic Disease Working Group) and the Association for European Paediatric and Congenital Cardiology (AEPC). A group of experts from 12 different centres and 8 different countries participated in this effort. This document reviews four main subjects, namely, (i) imaging of the aorta at diagnosis and follow-up, (ii) recommendations on medical treatment, (iii) recommendations on surgical treatment, and (iv) recommendations on sport participation.

## Graphical Abstract

**Graphical Abstract:**
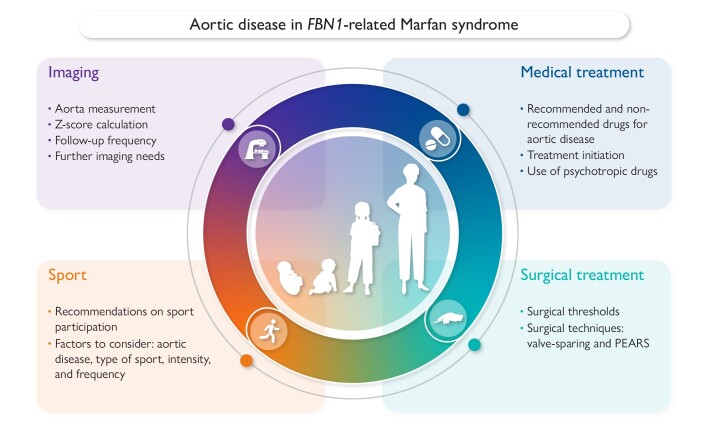
Overview of the four main topics addressed in this joint statement and the most important questions addressed within each topic. This document focuses on follow-up and treatment of aortic disease in children with Marfan syndrome and carrying a pathogenic variant in the fibrillin-1 (*FBN1)* gene. PEARS, personalized external aortic root support.

## Preamble

This joint statement document by the paediatric subgroup of the European Reference Network of Vascular Diseases (VASCERN) and the Association for European Paediatric and Congenital Cardiology (AEPC) represents their first collaborative effort. It reviews current knowledge on the follow-up and treatment of children with Marfan syndrome (MFS), providing recommendations based on clinical evidence or on expert opinion. The level of final agreement for each statement has been added to the corresponding table.

### Expert panel organization

The lack of standardized care for children with MFS across Europe prompted the development of this document. The expert panel consisted of 14 experts from 12 centres and 8 countries. Members of VASCERN's Heritable Thoracic Aortic Disease (HTAD) Working Group or the AEPC's Genetics, Basic Science and Myocardial Disease Working Group participated.

### Methodology

**Figure 1:**
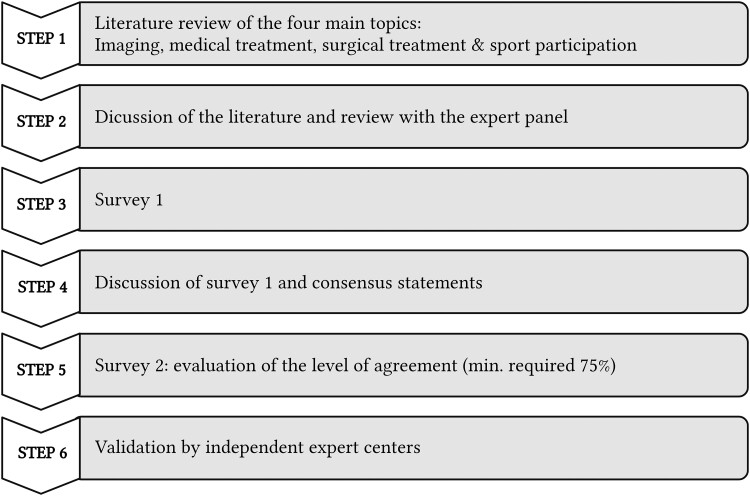
Stepwise process of the creation of the consensus document. The four main topics treated were (i) imaging of the aorta, (ii) medical treatment, (iii) surgical treatment, and (iv) sport participation. Literature search was mainly performed in PubMed and Web of Science. The online discussions were followed by dedicated surveys to evaluate the level of agreement to each statement. If a statement had <75% agreement, a new round of online questions was performed based on the former feedback of all group members. This process was repeated until statements were formulated with a minimum of 75% agreement. The document was validated in a final step by several independent expert centres as indicated in *Acknowledgements*

Monthly online discussions, held from November 2021 to May 2023, reviewed the current published literature focused on children with MFS and single-centre practices on four main topics: (i) imaging of the aorta, (ii) medical treatment, (iii) surgical treatment, and (iv) sport participation. Literature search was mainly performed in PubMed and Web of Science. After the online calls, surveys gauged agreement on statements, with rounds repeated until achieving ≥75% agreement. To validate the formulated statements, the consensus document was reviewed by several independent experts who are listed in the *Acknowledgements* section. The sequential process is shown in *Figure 1*.

### Scope

This document aims to assist paediatric cardiologists in the follow-up and treatment of children with MFS related to pathogenic variants in fibrillin-1 (*FBN1*), focusing on aortic disease. It excludes mitral valve disease and other cardiovascular problems like cardiomyopathy, as well as other non-*FBN1*–related HTAD.

## Introduction: Marfan syndrome

Marfan syndrome is a connective tissue disorder caused by pathogenic variants in the *FBN1* gene encoding the extracellular matrix (ECM) protein fibrillin-1.^1^ Fibrillin-1 is ubiquitous throughout the organism, thereby accounting for the diverse spectrum of manifestations associated with this condition. The diagnosis is usually clinical and based on the revised Ghent nosology.^2^ Clinical diagnosis, however, might be challenging because systemic features can be subtle or absent in very young children.^3^ Some children might also be diagnosed through predictive genetic testing, following the identification of an affected family member.

### Clinical spectrum

Marfan syndrome is a highly penetrant condition that demonstrates substantial intra- and interfamilial variability. Part of this variability might be explained by the underlying pathogenic variant.

#### Early-onset Marfan syndrome

Early-onset MFS (eoMFS) is a severe form of the disease.^4^ The definition remains however unclear and not recognized internationally. Individuals with eoMFS usually present specific marfanoid characteristics at the time of birth including camptodactyly, arachnodactyly, joint contractures, muscle hypoplasia, and loose skin.^4–6^ They frequently develop significant cardiorespiratory compromise, including congenital emphysema and atrioventricular valve regurgitation leading to early mortality. These individuals often have *de novo* pathogenic variants (missense and in-frame deletions) in *FBN1* clustered within exons 24–32.^7^ This document excludes patients with eoMFS, where cardiac failure is the most important cardiovascular feature and determinant for the outcome.

#### Classic Marfan syndrome

Cardinal manifestations in classic MFS involve the ocular, skeletal, and cardiovascular systems.^8^ Cardiovascular features in children include dilatation of the aorta at the level of the sinuses of Valsalva, mitral valve prolapse with or without regurgitation, tricuspid valve prolapse, and proximal pulmonary artery enlargement. Aortic root dissection is unusual in children but might occur in adolescents. Severe and prolonged left-sided valve regurgitation can predispose to left ventricular dysfunction and occasionally heart failure.^9^ Aortic dilatation at distal sites, primary cardiomyopathy, and ventricular arrhythmia leading to sudden cardiac are less common, yet noteworthy cardiovascular complications, reported mainly in adults.^10–12^

Skeletal manifestations include bone overgrowth and joint laxity; disproportionately long extremities for the size of the trunk (dolichostenomelia); overgrowth of the ribs that can push the sternum in (pectus excavatum) or out (pectus carinatum); and scoliosis that ranges from mild to severe. Ocular findings include myopia (>50% of affected individuals); ectopia lentis (seen in ∼60% of affected individuals); and an increased risk for retinal detachment, glaucoma, and early cataracts.^8^

### Genetics of Marfan syndrome

In the early 1990s, pathogenic variants in *FBN1* were identified as the cause of MFS.^1,13^ Pathogenic variants predisposing to MFS are distributed throughout the gene and are mostly private.^8^ Missense variants typically disrupting the repetitive calcium-binding epidermal growth factor (cb-EGF)–like domains are the most common type of disease-causing variants.^14^ Approximately 10% of the disease-causing variants disrupt canonical splice donor or acceptor sites and cause splicing errors, which can lead to in-frame deletion of an entire cb-EGF–like domain. Variants leading to splicing errors can also cause a frameshift in translation and, along with small insertions, deletions, and stop codons, lead to haploinsufficiency. Fibrillin-1 haploinsufficiency is the cause of MFS in 30%–40% of cases.^15,16^ Up to 7% of MFS pathogenic variants are large or complete deletions of *FBN1*.^15^

About 25% of *FBN1* pathogenic variants are *de novo*^17^ with gonad mosaicism, in which unaffected parents harbour the pathogenic variant in some of their germline cells and somatic mosaicism also being observed.^18,19^

The most consistent and robust genotype–phenotype association exists with *de novo* missense pathogenic variants in exons 24–32 in eoMFS.^7^ Additional correlations include pathogenic variants disrupting cysteine in patients with ectopia lentis^7,20^ and haploinsufficiency variants associated with more severe skeletal features.^16,20^ Cardiovascular genotype–phenotype correlations are more debated: pathogenic variants leading to haploinsufficiency and variants disrupting cysteine residues seem to be associated with a more severe phenotype.^16^ Conversely, missense variants introducing a cysteine residue tend to associate with milder cardiovascular features.^16^

### Diagnosis in paediatric patients

Early diagnosis is crucial for implementing appropriate surveillance and intervention strategies to optimize outcomes in affected individuals. In individuals with a family history of MFS and a known pathogenic variant, genetic testing can be offered. Asymptomatic children diagnosed through cascade screening should be offered comprehensive evaluation every 1–2 years.

When clinical suspicion is high but family history is negative, a comprehensive evaluation including cardiovascular, skeletal, and ocular assessments should be performed to evaluate the clinical features included on the revised Ghent nosology.^2^ Patients (almost) fulfilling these criteria can be offered genetic testing. Testing may be deferred in very young children if only some skeletal features are present; instead, a follow-up evaluation should be offered.

### Aortic disease in Marfan syndrome

The aortic root is the initial segment of the aorta and comprises the aortic valve annulus, the sinuses of Valsalva, and the sinotubular junction (ST-junction). Aortic root dilatation is the hallmark of MFS, present in 75%–80% of affected children.^3^ The predilection of the aortic root to dilate is determined by a combination of haemodynamic and structural factors. On one hand, the proximal part of the aorta bears the cyclic pressure load from the left ventricular ejection, rendering it more susceptible to dilatation. Conversely, the elastin content at the level of the sinus is higher than in other parts of the arterial tree,^21^ and therefore, diseases like MFS affecting elastogenesis confer a higher risk of dilatation at this site.

Aortic root dilatation can be typically diagnosed by echocardiography, with computed tomography angiography (CTA) and magnetic resonance imaging (MRI) providing more accurate measurements. In adult patients, prophylactic aortic root replacement is usually indicated when the diameter reaches 50 mm with earlier surgical intervention considered at 45 mm for rapid aortic growth, progressive aortic valve regurgitation, family history of dissection, or pregnancy desire.^22,23^

If the dilatation advances and surgery is not performed, dissection or rupture may occur. Typically, MFS patients will have a dissection at the level of the proximal aorta (type A dissection), but dissection at distal sites (type B dissection) has also been described in adult cohorts.^10,11^ While increased aortic diameter correlates with increased risk of dissection,^24^ some patients experience dissection below surgical thresholds.^24^ Some factors like dilatation of the ST-junction, rapid aortic growth, or male sex^25–27^ have been associated with a higher incidence of aortic root replacement but not necessarily with a higher rate of dissection. Aortic stiffness and aortic and vertebral tortuosity have been associated with worse cardiovascular prognosis.^28–30^

## Imaging of the aorta

Accurate and reproducible aortic measurement is crucial for the diagnosis, surveillance, and management. Additionally, surgical planning relies on quantification of absolute diameters. There are no established recommendations on imaging and surveillance in children with MFS except for Canadian guidelines which attempt a time frame for baseline assessment and 6-monthly monitoring with 2D transthoracic echocardiography (2D-TTE), without actually prescribing clear intervals for CTA/MRI.^31^ It has been common practice to adapt adult guidelines in the absence of a more tailored and children centred approach.^22^

### Echocardiography: technical aspects and calculation of the *z*-score

2D-TTE is the imaging modality of choice for measuring proximal aortic segments and is commonly used for initial assessment and surveillance of aortic dilatation.

#### How to measure the aorta

**Figure 2:**
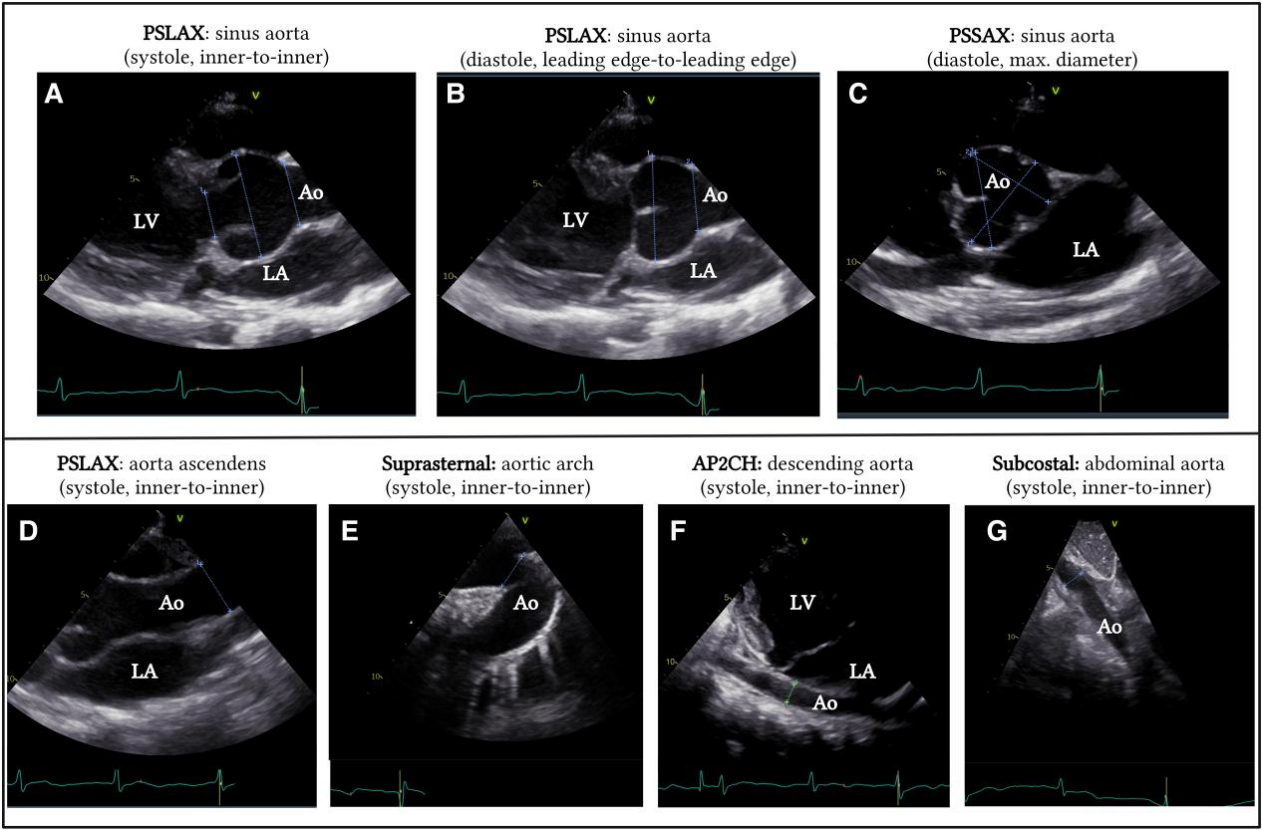
Echocardiographic imaging of the aorta at different levels. *A* Parasternal long-axis view of the aortic annulus, sinus of Valsalva, and sinotubular junction measured in systole using the inner-to-inner method. Measurements should be taken perpendicular to blood flow. *B* Parasternal long-axis view of the sinus of Valsalva and sinotubular junction measured in diastole using the leading edge-to-leading edge method. *C* Parasternal short-axis view of the aortic valve measured in diastole and using the largest aortic diameter. *D* Parasternal long-axis of the ascending aorta, measured in systole at the level of the right pulmonary artery, using the inner-to-inner method. *E* Suprasternal view of the aortic arch measured in systole between the truncus brachiocephalicus and the left carotid artery, using the inner-to-inner diameter. *F* Modified apical two-chamber view of the descending aorta measured at the level of the left atrium in systole using the inner-to-inner method. *G* Subcostal view of the abdominal aorta at the level of the liver, measured in systole using the inner-to-inner diameter. Ao, aorta; AP2CH, apical two-chamber view; LA, left atrium; LV, left ventricle; PSLAX, parasternal long-axis view; PSSAX, parastenal short-axis view

There is no universally accepted way of measuring aortic root diameter. According to the American Society of Echocardiography (ASE) guidelines for paediatric patients, measurements should be taken perpendicular to the long axis of the aorta at specific anatomic landmarks.^32^ A 2D image of the aorta should be recorded in the parasternal long-axis (PSLAX) view. Aortic diameters at the annulus, the sinuses of Valsalva, ST-junction, and ascending aorta at the level of the right pulmonary artery should be measured (*Figure 2A, B,* and *D*). All measurements should be obtained in systole, from inner edge to inner edge, at the broadest diameter. The highest values are used for *z*-score calculation. Both raw averaged values of aortic diameters and the corresponding *z*-scores should be reported.

In contrast to the paediatric guidelines, the ASE and the European Society of Cardiology (ESC) guidelines for adult patients recommend the use of the leading edge to leading edge method in diastole.^33,34^ While this working group advocates for using the inner-to-inner diameter in systole, recognized as the most validated method in paediatric patients, some expert centres prefer adult recommendations. This approach ensues consistent follow-up across the lifespan and aligns better with CTA and MRI measurements.^35^ Studies in children and adults with MFS, as well as healthy controls, demonstrated that standard systolic and diastolic echocardiographic measurements of the aortic root are comparable and show good interobserver agreement.^36–39^ Maintaining consistency in the chosen method is the most important aspect, regardless of the specific approach.

By using the PSLAX view, the maximum aortic root diameter is determined by measuring the distance between the right and non-coronary cusps. However, due to complex, non-cylindrical geometry of the aortic root, this might not be the largest aortic root diameter. Measurements from the parasternal short axis may help identify significant aortic root asymmetry; however, cross-sectional imaging with CMR or CTA is more reliable (*Figure 2C*).^34^

In contrast to adults, visualization of the distal parts of the aorta in children using 2D-TTE is usually feasible. The suprasternal view allows visualization of the aortic arch and proximal descending aorta, while the modified apical two-chamber view and the subcostal view provide views of the descending and abdominal aorta, respectively (*Figure 2D–G*).^34^

#### Applicability of the different *z*-scores

*Z*-scores based on body surface area (BSA) play a crucial role in interpreting aortic diameters. Despite numerous published nomograms for aortic measurements, there is no consensus on the preferred one. Nomograms lead to different *z*-scores due to variations in measuring methods, BSA normalization technique, and diversity of the study population.^40^ The Haycock formula for BSA is often favoured over the Du Bois and Du Bois formula for its accuracy in younger children.^41^ An overview of the different nomograms is presented in *Table 1*.^42–49^ Among the available options, the Lopez *et al.* method^42^ stands out for its inclusion of a large and diverse population (*N* = 3215), utilizing the inner-to-inner edge method in systole along with the Haycock formula to calculate BSA. However, it is worth noting that this method may yield higher *z*-score calculation compared with others.^40^ This needs to be taken into consideration, particularly in diagnostic settings.

**Table 1:** Overview of the different methods for calculating *z*-scores in children

Reference	TTE method	Population	Year
Roman *et al.*^44^	M-mode and 2D-TTE Leading to leading edge, diastole	*N* = 187 total *N* children: 52 (30 days–15 years) Healthy individuals	1989
Colan *et al.*^45^ (Sluysmans *et al*.)	2D-TTE Inner to inner edge, systole BSA method: Haycock	*N* = 496 (1 d–20 yr) Healthy individuals	2005
Warren *et al.*^46^ *(Halifax method)*	2D-TTE Inner to inner edge, systole BSA method: Boyd	N = 88 (1 d–18 yr) Only children with BAV	2006
Pettersen *et al.*^47^ *(Detroit method)*	M-mode and 2D-2D-TTE Inner to inner edge, systole	*N* = 782 Healthy children	2008
Gautier *et al.*^48^	2D-TTE Leading to leading edge, diastole BSA method: Du Bois and Du Bois	*N* = 353 (2–18 yr) Healthy French children	2010
Campens *et al.*^43^	2D-TTE Leading to leading edge, diastole BSA method: Du Bois and Du Bois	*N* = 849 total *N* children: 80 (1–15 yr)	2014
Lopez *et al*.^42^ *(Boston method)*	2D-TTE Inner to inner edge, systole BSA method: Haycock	*N* = 3215 (1 d–18 yr) Healthy North American children	2017
Cantinotti *et al.*^49^	2D-TTE Inner to inner edge, systole BSA method: Haycock	*N* = 1151 (1 d–18 yr) Healthy Italian children	2017

2D-TTE, 2D transthoracic echocardiography; BAV, bicuspid aortic valve; BSA, body surface area; d, days; yr, years.

For measurements taken in diastole, the Campens nomograms are preferred^43^ given its validation across both paediatric and adult populations.

#### Recommendations for echocardiographic examination (*Table 2*): specific supporting text

- The aorta should be measured at seven different levels (annulus, sinus, ST-junction, ascending, arch, and descending and abdominal aorta). The preferred method is using the inner-to-inner edge in systole, according to the ASE recommendation for paediatric patients.
- The Lopez *et al.* method for calculating *z*-scores is recommended.
- When measuring the aortic sinus and ascending aorta using the leading edge to leading edge in diastole, the Campens nomograms are recommended for *z*-score calculation.
- Given the variability of the *z*-scores depending on the method used, it is recommended to consistently use the same method for follow-up comparison.
- Yearly 2D-TTE surveillance is recommended in children with MFS. Children with large aortas, showing accelerated aortic growth (>5 mm/year), having additional cardiac involvement or on the starting phase of medical treatment, might benefit from a more frequent assessment.

### Magnetic resonance imaging

#### Magnetic resonance imaging: technical and practical aspects

While 2D-TTE serves as the standard modality for aortic measurement, cross-sectional imaging through MRI offers superior accuracy, especially in cases of chest wall deformity or with asymmetric aortic roots.^50^ Magnetic resonance imaging is preferred over CTA, because it does not use ionizing radiation and can often be performed without intravenous contrast. This is particularly important for lifelong surveillance in children to prevent cumulative radiation exposure. However, MRI spatial resolution is inferior to CTA and acquisition time much longer; for these reasons, CTA remains the gold standard in situations requiring rapid assessment of the aortic wall integrity. Metallic implants (even when MRI compatible) can significantly degrade the image quality. Single-institution MRI protocols may vary, and paediatric recommendations are lacking.

**Table 2:** Summary of the different recommendations and level of agreement

	LoA (%)
**Imaging**	
2D-TTE is the imaging technique of choice for diagnosis and surveillance	100
The aorta should be measured at the seven different recommended levels	100
The inner-to-inner edge method in systole is the preferred method to measure the aorta in 2D-TTE	92
The calculation of the *z*-scores for 2D-TTE measurements according to Lopez *at al.* may be considered as a method of choice	92
Alternatively, the leading edge-to-leading edge in diastole method can be considered. In this case, calculating *z*-scores according to the Campens nomograms is recommended	100
2D-TTE should be performed in each child with MFS at diagnosis; yearly examination thereafter is recommended	92
In patients with large aortic diameters (≥45 mm) or rapid aortic growth (>5 mm/yr with increase in *z*-score of >1 SD) biannual surveillance is recommended	92
A biannual 2D-TTE can be considered during titration of medication or if clinically indicated (e.g. valvular disease)	92
MRI should be preferred over CTA for surveillance in children to limit radiation exposure	83
The calculation of the *z*-score for MRI measurements according to Kaizer *et al*. is recommended	92
CTA should be considered as first choice if MRI is not tolerated or would not allow acquisition of diagnostic images, in specific instances for surgical planning (i.e. PEARS), and in the suspicion of an acute aortic event	100
Cross-sectional imaging (MRI/CTA) can be offered to all children with MFS during adolescence, when no sedation is required, to gather baseline aortic measurements	83
In children with (almost) normal aortic diameter, appropriate visualization, and no asymmetry of the aortic valve, cross-sectional imaging (MRI/CT) can be deferred to an adult age	100
Cross-sectional imaging (MRI/CTA) can be considered at regular intervals according to clinical progression	83
Cross-sectional imaging (MRI/CTA) can be considered at an earlier age or interval (even if sedation or general anaesthesia is needed) in circumstances where 2D-TTE does not provide accurate data, if approaching surgical threshold, or in presence of accelerated aortic growth (>5 mm/year with increase of *z*-score of >1 SD)	83
Cross-sectional imaging (MRI/CTA) should be considered at an earlier age or interval if indicated to assess or monitor other lesions (i.e. valvular and/or cardiac function and shunts)	83
Neck-to-pelvis MRI may be considered in all patients at first MRI examination	83
MRI of cerebral arteries is not required in children with MFS	100
**Medical treatment**	
In children with MFS and aortic dilatation, medical treatment with a BB or ARB at maximally tolerated doses is recommended	83
Treatment can be considered in children with MFS and no aortic dilatation especially in the presence of additional risk factors	100
Combined therapy with BBs and ARBs should be considered if fast progression is observed during surveillance (5 mm/yr) or if aortic diameter > 40 mm or *z*-score *>* 5.	83
ACE-I can be considered if BBs and ARBs contraindicated	92
Calcium channel blockers should be avoided as they might increase risk of acute aortic events	100
**Surgical treatment**	
Surgery is recommended in children when the aortic root diameter reaches 50 mm.	100
Surgery can be considered at 45 mm when some additional risk factors are associated such as family history of aortic dissection, rapid annual growth rate > 5 mm/yr, and when concomitant valve surgery is indicated	83
Aortic valve–sparing surgery is preferred to the aortic valve replacement technique	92
**Sport participation**	
The following aspect should be taken into consideration when recommending sport participation: type of sport, the frequency and the intensity of the sport, and the severity of the aortic disease	100
Sports at competitive level are not recommended except for skills, low-intensity sports	83
In children ≤10 yrs, any recreational sport can be considered without restriction, but the possibility of restricting a sport with age should be discussed	100
In children >10 yrs, power sports are not recommended	92
In children >10 yrs and an aortic root *z*-score < 3: skill, mixed, or endurance sports can be considered at a recreative level, at any intensity	92
In children >10 yrs and an aortic root *z*-score ≥ 3 or ≥ 40 mm: skill, mixed, or endurance sports can be considered at a moderate level (defined as being able to hold a conversation with a friend during exercise)	92

2D-TTE, 2D transthoracic echocardiography; ACE-I, angiotensin-converting enzyme inhibitors; ARBs, angiotensin receptor blockers; BBs, beta-blockers; CTA, computed tomography angiography; LoA, level of agreement; MFS, Marfan syndrome; MRI, magnetic resonance imaging; PEARS, personalized external aortic root support.

For the isolated aortic root assessment, non-contrast MRI sequences are recommended. Electrocardiogram (ECG)-gated imaging decreases motion artefacts, while free-breathing black-blood sequences, 3D steady-state free precession (SSFP), and cine imaging usually allow to obtain a 3D data set for precise and repeatable measurements.^51,52^

Although less common in MFS than in other connective tissue diseases, adolescents and young adults may present vascular problems distal to the ascending aorta.^53,54^ Tortuosity of the neck vessels is well recognized, and an established predictor of outcome in children with MFS,^34^ and therefore a neck-to-pelvis angiography assessment, may be warranted. This usually requires non-contrast sequences like *time-of-flight* for neck vessels and axial and coronal *true fast imaging with steady-state precession* (TruFISP) together with contrast-enhanced (single bolus) magnetic resonance angiography (MRA).

When assessing aortic measurements on MRI and CTA, nomogram issues persist, with adult recommendations applied to older children and adolescents.^22^ Kaiser and colleagues^55^ published a data set to calculate *z*-scores for thoracic aorta diameters derived from MRI in children. Despite limitations such as reliance on contrast-enhanced angiography and lack of ECG gating imaging, these are the only nomograms offering a method for *z*-score calculation in paediatric patients.

#### Recommendations for magnetic resonance imaging (*Table 2*): specific supporting text

- Cross-sectional imaging using MRI is preferred over CT to limit the cumulative radiation exposure. Computed tomography angiography remains the gold standard for surgical planning, in the acute setting, and when MRI is not tolerated or would yield inconclusive results.
- Magnetic resonance imaging screening can be considered when children can tolerate an awake scan, usually around age 10. In children without clear aortic root dilatation and symmetric aortic valve on 2D-TTE, deferring MRI scan until adulthood can be justified.
- Regular surveillance with cross-sectional scans can be considered every 5 years especially in patients with large diameters and/or asymmetric aortic valves. Magnetic resonance imaging surveillance should align with clinical needs.
- Early or more frequent screening is recommended when 2D-TTE fails to provide adequate images for safe surveillance (poor acoustic window, chest wall deformity, asymmetric aortic root, etc.), when approaching surgical threshold, or in the presence of accelerated aortic growth (>5 mm/year with a significant increase in *z*-score of at least 1 SD).
- Neck-to-pelvis MRI can be considered at baseline to assess tortuosity of neck vessels, a well-recognized and an established predictor of outcome.
- Magnetic resonance angiography of cerebral vessels is not required in children with MFS due to the likely low prevalence of cerebral aneurysm and lack of established management protocols.^56^

### Cardiac computed tomography angiography

#### Computed tomography angiography technique

Computed tomography angiography can be useful in imaging the aorta in patients with MFS, especially in those patients in whom cross-sectional imaging is indicated and who are unable to tolerate an MRI scan without prolonged sedation or general anaesthesia.^22^ Using third-generation CT scanners with dual sources of radiation/ultrawide detectors with a high temporal resolution (66–75 ms), scans can be obtained in paediatric patients within 1–3 s, thereby enhancing successful acquisition rates.^57^ While oral sedation may be required for children under 5 years old to facilitate a free-breathing scan, those over 6 years often tolerate awake scans well. To minimize radiation exposure, scans should be acquired using prospective ECG gating, employing techniques like the step-and-shoot, high-pitch spiral acquisition with prospective ECG triggering on dual-source scanners, or target ECG-gated method on scanners with ultrawide detectors.^58^ The scan should be acquired in end-systole, using a relatively narrow window of acquisition to minimize radiation exposure. Using these low-radiation dose techniques, the field of view can be increased to cover the entire length of the thoracic aorta if required.

Computed tomography angiography serves as a valuable tool for procedure planning in paediatric patients nearing the surgical threshold and is particularly relevant for personalized external aortic root support (PEARS) planning where the spatial data from the high-resolution CT images are used to create a computer-aided design model from which a replica of the individual aorta is made by rapid prototyping.

#### Recommendations for computed tomography angiography (*Table 2*): specific supporting text

- Computed tomography angiography should be considered over MRI in children who cannot tolerate MRI without prolonged sedation or anaesthesia.
- Computed tomography angiography should be considered as a first imaging option in case of suspicion of an acute aortic event.
- Computed tomography angiography should be considered before surgical planning, especially in those children candidate for PEARS procedure.

## Medical treatment

### Different drugs used in treatment

If untreated, individuals with MFS may present earlier progressive aortic root dilatation.^59^ Improved survival in MFS over recent decades is attributed to better familial screening, regular surveillance, prophylactic medical treatment, and timely surgery.^60,61^ While it is clear that the arterial wall composition and biomechanical characteristics are altered and responsible for increased fragility, there are no reliable biomarkers to predict aortic events. The best predictor remains aortic root dilatation, and therefore, treatment has aimed at slowing aortic root growth. High blood pressure needs to be aggressively addressed, but it is uncommon in these young patients. Beta-blockers (BBs) are commonly used to slow aortic root growth by lessening strain on the arterial wall, yet their impact on clinical endpoints such as aortic dissection and mortality lacks robust evidence. Several observational studies and only one clinical trial have evaluated the effectiveness of the chronic use of BBs in patients with MFS,^62^ and the results have been conflicting. Nevertheless, the use of BBs is considered standard of care in patients with MFS. The most widely studied BBs are atenolol and propranolol,^62–71^ but bisoprolol and metoprolol have also been used in several studies.^72,73^ Research has more recently focused on agents which could affect the natural history of the disorder by manipulating the signalling pathway of the diseased aorta itself. In particular, the use of angiotensin receptor blockers (ARBs), especially losartan, has been adopted by many.^69,70,72,74–77^ Irbesartan and valsartan have also been used in several studies.^73,78,79^ ARBs attenuate the transforming growth factor beta (TGFβ) activity, possibly leading to a reduction in ECM degeneration in the vessel wall, in addition to having a blood pressure–lowering effect. While losartan's efficacy was significant in animal models,^80^ its effect in human is less pronounced.^81^ Several groups have investigated the effects of ARBs in humans compared with or in addition to BBs, obtaining variable results.^69,70,72,73,76,77^ A recent meta-analysis including 1442 patients (children and adults) showed a positive effect of ARB therapy in reducing aortic root *z*-score, similar to that of BBs.^82^ Accordingly, it appears both classes of medicines are effective in slowing down the aortic root growth, and combination therapy appears more effective than monotherapy in children with MFS. Current ACC/AHA guidelines recommend use of either BBs or ARBs or a combination of both at maximally tolerated doses (*Table 3*).^22^ European guidelines recommend use of BBs and consider ARBs as an alternative treatment.^23^ Evidence to substantiate the effectiveness of angiotensin-converting enzyme inhibitors (ACE-I) in attenuating aortic dilatation is currently lacking.^68^ They can be considered in patients who have contraindications to BBs and ARBs. Use of calcium channel blockers has been linked to an increase in acute aortic events, and therefore, they should not be used in MFS.^83^

**Table 3:** Recommended dosage of oral therapy in children with Marfan syndrome

Drug name	Dosage/kg/day	Max. dosage
**Beta-blocker**		
Atenolol	1–4 mg/kg/d	250 mg
Propranolol	0.75–3 mg/kg/d	160 mg
Metoprolol	1–2 mg/kg/d	200 mg
Bisoprolol	0.05 mg/kg/d	10 mg
**Angiotensin receptor blocker**		
Losartan	0.7–1.4 mg/kg/d	50 mg ≤50 kg 100 mg >50 kg
Irbesartan	1–2 mg/kg/d	150 mg ≤50 kg 300 mg >50 kg
Valsartan	1–4 mg/kg/d	80 mg ≤35 kg 160 mg between 35–80 kg 320 mg ≥80 kg
**ACE-I**		
Enalapril	0.1–1 mg/kg/d	20 mg
Captopril	0.3–6 mg/kg/d	100 mg

The optimal timing for initiating treatment is debated with certain studies indicating more favourable outcomes with early or prolonged therapy, particularly in children with existing aortic root dilatation.^67,69^ Overall, current available evidence supports initiation of prophylactic medical therapy in children with MFS if (mild) aortic root dilatation is present and the benefit of starting treatment before dilatation occurs; it needs to be individually considered and discussed with the child's parents or guardians. Given that aortic root dilatation in MFS has incomplete penetrance, treating all patients would lead to unnecessary lifelong treatment of about 20%.

### Recommendations for medical treatment (*Table 2*): specific supporting text

- The expert group recommends starting medical therapy when the aorta *z*-score reaches 2 or higher.
- Medical treatment can be considered in patients with a confirmed diagnosis and normal aortic size with specific indicators of a more aggressive vascular phenotype: arterial tortuosity, carriers of a truncating variant, or a variant causing loss-of a cysteine residue in a cb-EGF–like domain and family history of early dissection.^16^
- Particular attention should be given to relative contraindication to treatment: for BBs, asthma, hyperinsulinism, or challenging glucose levels and for ARBs, teenage girls without contraception or infants below the age of 1 year. Adequate monitoring of potential side effects should be established.
- Combination therapy is suggested to further reduce aortic growth rate and is commonly prescribed when there is at least moderate dilatation (aortic root diameter ≥ 40 mm or *z*-score ≥ 5) or fast aortic growth rate progression (>5 mm/year with a significant increase in *z*-score of +1 SD/year).
- Medications should be titrated to the highest tolerated dose to achieve the desired effect of slowing dilatation. Effects can be titrated on haemodynamic responses like reduction of baseline heart rate as proposed by some authors.

### Psychotropic drugs used for the treatment of attention-deficit/hyperactivity disorder and related conditions

While patients with MFS typically have normal intellectual and gross motor development, up to 50% present with neuropsychological deficits, including attention-deficit/hyperactivity disorder (ADHD).^84^ Data from the Pediatric Heart Network Marfan trial revealed that ADHD significantly impacts health-related quality of life in children and young adults with MFS.^84^ The US Food and Drug Administration–approved drugs for treatment of ADHD include stimulant drugs (amphetamine and methylphenidate) and non-stimulant drugs (atomoxetine, clonidine, and guanfacine).^85^ The European Medicines Agency has not approved the use of clonidine but has a waiver for the use of prolonged clonidine release for paediatric patients.^86^ Stimulant drugs and atomoxetine may produce a mild increase in heart rate and blood pressure and could delay ventricular repolarization. Conversely, clonidine and guanfacine may reduce heart rate and blood pressure.^85^ Treatment decisions for ADHD in children with MFS should be made case-by-case. Non-stimulant drugs are considered safer but sometimes less-effective. When stimulant drugs are needed, usual caution and control of the heart rate and blood pressure as per standard guidance is warranted with cessation of the drug if hypertension is detected.

## Surgical management

### When to consider surgical replacement

Even though medical treatment can decrease the rate at which the aorta dilates and postpone the timing of surgery, aortic root intervention is the only effective way to prevent dissection. In contrast to surgery in urgent settings when a dissection has occurred, prophylactic surgery has a low mortality rate.^87,88^ Therefore, preventive aortic root intervention has become the standard of care.

The decision for surgical intervention is based on weighing the operative risk against the risk of aortic dissection, influenced by centre expertise, surgical technique, and the need for concurrent valve surgery.^89^ In-hospital mortality for elective surgery in experienced centres is estimated to be lower than 1%.^89–92^ Young children, however, have a higher rate of re-intervention, related to increase in size and disease progression.^92–94^

Scattered case reports exist of aortic dissection or rupture under the age of 10.^93,95,96^ Data from large national databases point at most of dissections starting to occur during adolescence. Approximately 0.5% of all dissections in MFS patients occur in this age category.^27,88,89,97^

There are no specific thresholds for preventive aortic root surgery in children with MFS. Current indications are mostly based on absolute aortic root diameters, annual growth rate, and the indication for concomitant valve surgery. The majority of the centres follow the existing adult AHA/ESC guidelines.

### Surgical techniques

A paramount consideration is the diameter of the native aortic valve which should be able to accommodate a prosthesis adequate for an adult body size. An annulus diameter of ≥22 mm, measured on echocardiography in PSLAX inner-to-inner diameter, is considered adequate.^98^

Several techniques to replace the aortic root have been used throughout the years. The dilated aortic root might be replaced alone (aortic-sparing surgery) or combined with the aortic valve (aortic valve replacement surgery). As in adults, the most widely accepted option in children is the valve-sparing root replacement technique, a durable repair achievable with low operative mortality.^99–101^ Within valve-sparing techniques, three modalities can be distinguished: the reimplantation technique (David V procedure), the remodelling technique (Yacoub procedure), and, more recently, the PEARS procedure. The reimplantation technique is preferred in adults due to the higher rate of re-intervention secondary to aortic regurgitation, observed in the remodelling technique.^92^ While the PEARS procedure shows promise with low mortality (<1% in adults), its adoption as standard care in children requires further long-term data validation.^102^

### Recommendations for surgical treatment (*Table 2*): specific supportive text

- Surgery is recommended in children when the aortic root diameter reaches 50 mm. It can be considered at 45 mm when some additional risk factors are present such as family history of aortic dissection at low diameter, rapid annual growth rate (>5 mm/year and *z*-score increase of +1 SD), and concomitant valve surgery indication.
- Aortic valve–sparing surgery is preferred to the aortic valve replacement because it avoids the need of lifelong anticoagulation.
- Within the aortic valve–sparing surgical modalities, the reimplantation technique is preferred.

## Exercise, recreational, and competitive sport

### Impact of exercise on the cardiovascular system

In individuals with MFS, strenuous exercise could exacerbate aortic dilatation and increase the risk of aortic dissection over time; therefore, historically, this concern led to recommending avoidance of intense physical activity.

**Figure 3:**
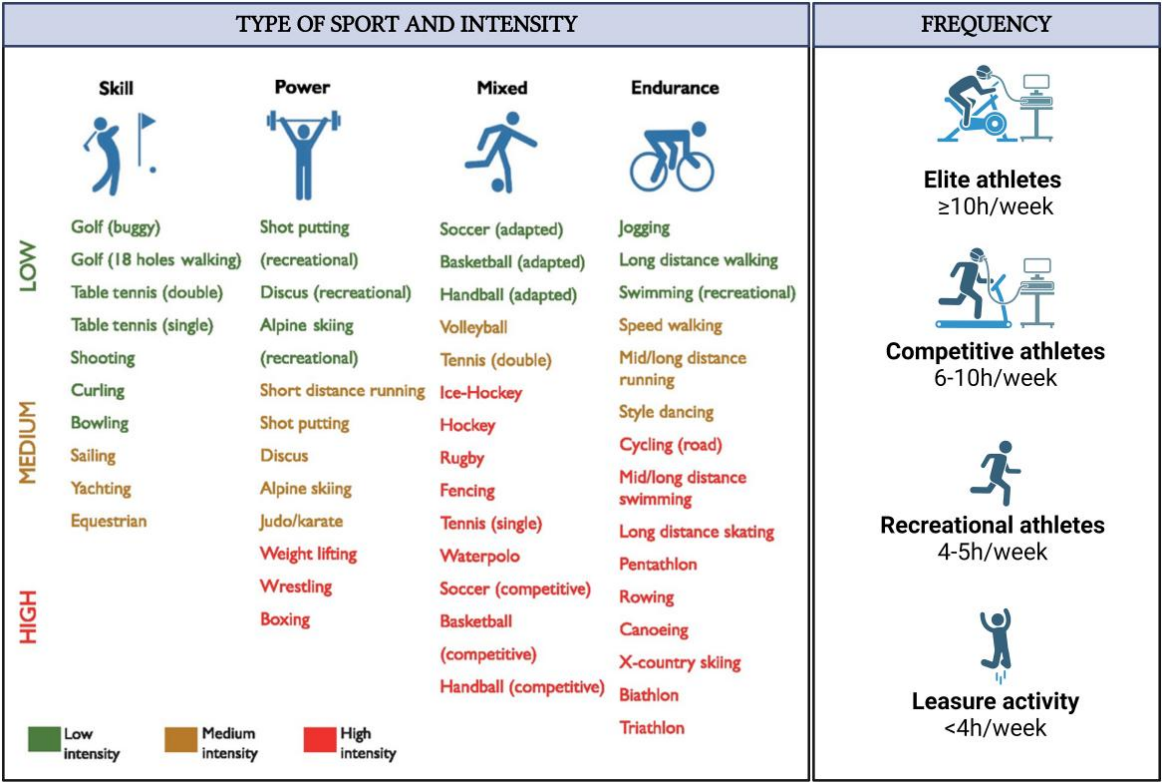
Differentiation of sports in relation to the predominant isotonic and isometric component, intensity, and frequency (adapted from the 2020 ESC guidelines on sport cardiology and exercise in patients with cardiovascular disease^103^)

The impact of exercise on the aortic wall varies depending on the type and intensity of the activity. Isotonic activities associated with an increase in cardiac output typically have a moderate effect on systemic blood pressure. In contrast, isometric activities cause a significant increase in diastolic and systolic blood pressure. Most sports combine isotonic and isometric components, which led to a new categorization into skill, power, mixed, and endurance sports^103^ (*Figure 3*). The frequency and intensity are also important and can be divided into elite, competitive, or recreational activity based on intensity and amount of training. As a rule, elite athletes train ≥10 h/week, competitive athletes 6–10 h/week, and recreational athletes 4–5 h/week, and <4 h/week is considered leisure sports.

Healthy children typically experience varying blood pressure levels during treadmill, with systolic blood pressure increases >160 mmHg in boys after age 14 while usually remains below 160 mmHg in girls regardless of age.^104,105^ No data are available on exercise-associated aortic growth. Athletes with bicuspid aortic valve (BAV) generally exhibit similar increase in aortic root and ascending aortic size to non-athletes with BAV.^106^ Furthermore, exercise-related aortic dissection is rare in healthy individuals,^107^ with the majority of cases involving adults during heavy lifting.^108^

Limited literature addresses the effect of exercise on aortic dilatation and dissection in patients with MFS. A recent prospective study suggests that a personalized home training programme using endurance and resistance exercise may be safe for adult patients with MFS with a maximum aortic diameter of 45 mm.^109^ Additionally, in mouse models of MFS, mild aerobic exercise appears to decrease elastin fibre fragmentation and slow the rate of aortic root dilatation, whereas no training at all or high-intensity training led to a worse aortic phenotype.^110,111^

### Recommendations for sport participation (*Table 2*): supporting text

Studies addressing sport participation are sparse, and no updated recommendations for children exist. Recommendations were therefore based on adult guidelines adjusted to the physiology of children. It is crucial to adopt a shared decision approach tailored to each child's circumstance when advising on exercise. Notably, additional restrictions related to ocular and skeletal problems are not addressed in our recommendation.

- Besides the severity of the aortic disease, the type, frequency, and intensity of the sport should be carefully evaluated with aortic imaging adapted accordingly.
- Competitive sport participation is not recommended except for skill, low-intensity sports as bowling, curling, and golf.
- In children ≤10 years, any recreational sport can be considered without restriction although the possibility age-related restriction should be discussed with the patient and the parents or guardians.
- In children >10 years, power sports are not recommended.
- In children >10 years and an aortic root *z*-score < 3, skill, mixed, or endurance sports can be considered at a recreational level, at any intensity.
- In children >10 years and an aortic root *z*-score ≥ 3 or diameter ≥40 mm, skill, mixed, or endurance sports can be considered at a recreational level, at a moderate intensity (defined as being able to hold a conversation with a friend during exercise).
- Similar considerations should be applied when advising patients on future job selection, as certain professions may entail significant physical demands.

## Summary

Aortic disease in MFS remains the main cause of morbidity and mortality in this group of patients. Although the incidence of aortic complications in children and adolescents with MFS remains low, early diagnosis and treatment can prevent severe problems in adulthood. The aim of this document is to provide guidance for the follow-up and treatment of children with MFS. Each patient should however be addressed through individualized counselling and treatment.

## Declarations

### Data Availability

No data were generated or analysed for or in support of this paper.
